# A comparison of KONAR and Amplatzer: are the p-values and reports of reintervention consistent?

**DOI:** 10.1186/s43044-026-00790-z

**Published:** 2026-10-30

**Authors:** Alper Akın

**Affiliations:** Department of Pediatric Cardiology, Dicle University, Diyarbakır, Turkey

Dear Editor,

I read the study by Taha et al., titled “Comparison of KONAR-multifunctional occluder and Amplatzer occluders in transcatheter closure of patent arterial ducts and ventricular septal defects,” in which the authors compared the KONAR-MFO and Amplatzer devices for the closure of PDA and VSD [1]. While reviewing the complication data, I noticed that some p-values did not match the reported numbers of patients and events.

Table 4 states that complete heart block developed in two of the 29 ADO-I patients who underwent VSD closure, while this complication was not observed in the 28 KONAR-MFO patients, and the p-value is given as 0.026. A 2 × 2 contingency table created with these numbers yields a p-value of 0.491 when analyzed using the two-way Fisher’s exact test. Similarly, while mild aortic insufficiency was not observed in the ADO-I group, it was reported in three patients in the KONAR-MFO group, with a p-value of < 0.001. The p-value calculated using the two-way Fisher’s exact test for this comparison is 0.112.

Due to the small number of events and cells containing zeros, the Fisher test is appropriate for these comparisons [2]. However, when the Pearson chi-square test is used without continuity correction, p-values of 0.157 and 0.070 are obtained, respectively. Therefore, the difference does not appear to stem solely from the choice of test. If the numbers in the table are correct, the reported statistical significance cannot be confirmed by these calculations. I believe the authors should clarify which comparisons the p-values were derived from and recheck the relevant analyses.

There is also an inconsistency between the text and the tables regarding re-intervention. In Tables 3 and 4, the “Re-intervention” row is listed as zero for all device groups; the results sections for the PDA and VSD subgroups also state that no patients required re-intervention. In contrast, the “Success rate and procedural complications” section of the discussion states that re-intervention was required in five patients (2.9%) and that the embolized devices were removed with a snare and larger devices were placed. Since the authors themselves refer to these interventions as “re-intervention,” it cannot be assumed that the discrepancy stems solely from distinguishing between rescue interventions performed during the procedure and subsequent interventions. It is necessary to clarify which report is accurate and to correct the text and tables accordingly. This inconsistency directly affects the interpretation of the results, given that reintervention was defined as one of the secondary endpoints in the study.

These points do not diminish the clinical significance of the complications. The fact that complete heart block or aortic insufficiency was observed in only one group should be taken into account; however, this observation is not the same as a statistically demonstrated difference between the devices. Clarifying the number of events, p-values, and the definition of reintervention will allow for a more accurate assessment of safety outcomes.

## Data Availability

No datasets were generated or analysed during the current study.
